# Microscope-Based Augmented Reality: A New Approach in Intraoperative 3D Visualization in Microvascular Decompression?

**DOI:** 10.7759/cureus.62417

**Published:** 2024-06-15

**Authors:** Levent Tanrikulu

**Affiliations:** 1 Neurooncology, Klinik Sonnenblick, University of Marburg, Marburg, DEU

**Keywords:** neuronavigation, augmented reality, 3d visualization, microvascular decompression, neurovascular compression

## Abstract

Neurovascular compression (NVC) syndromes such as trigeminal neuralgia (TN) are causally treated with microvascular decompression (MVD). Semiautomatic segmentation of high-resolution magnetic resonance imaging (MRI) data and constructive interference in steady state (CISS)/time-of-flight (TOF) sequences are utilized for the three-dimensional (3D) visualization of underlying causative vessels at the root entry zones of the relevant cranial nerves. Augmented reality (AR) of neurovascular structures was introduced especially in the resection of brain tumors or aneurysmatic operations. In this report, the potential feasibility of the implementation of microscope-based AR into the intraoperative microsurgical set-up of MVD was investigated. This article recommends the preoperative evaluation of 3D visualization besides the microscopical view of the surgeon. The implementation of multiple imaging data by AR into the operating microscope may afflict the experienced surgeon's view, which should be examined prospectively.

## Introduction

Microvascular decompression (MVD) is the most effective microsurgical treatment option in patients with intractable trigeminal neuralgia, hemifacial spasm, and glossopharyngeal neuralgia [1-7]. These neurovascular compression (NVC) syndromes are caused by aberrant arterial and/or venous vessel loops at the root entry zones [1-7].

With the routine diagnostic application of high-resolution magnetic resonance imaging (MRI) sequences such as constructive interference in steady state (CISS), T2-weighted single slab three-dimensional (3D) turbo spin echo (SPACE), and time-of-flight (TOF) angiography, the underlying causative vasculature can be segmented by image processing methods, and individual 3D visualization representations can be generated and presented during the preoperative and intraoperative set-up of MVD [1-2].

Neuronavigation and augmented reality (AR) have been introduced to several neurosurgical procedures, especially in tumor resections and spinal and vascular procedures [8-13]. AR is technically enabled by introducing optical information of segmented anatomical objects into the optical image of the operating microscope [14-17]. Several studies dealt with imaging and image postprocessing of NVC syndromes, while intraoperative AR has not been thoroughly examined in MVD. This article deals with the feasibility and potential implementation of AR into the intraoperative procedure of MVD.

## Technical report

**Figure 1:**
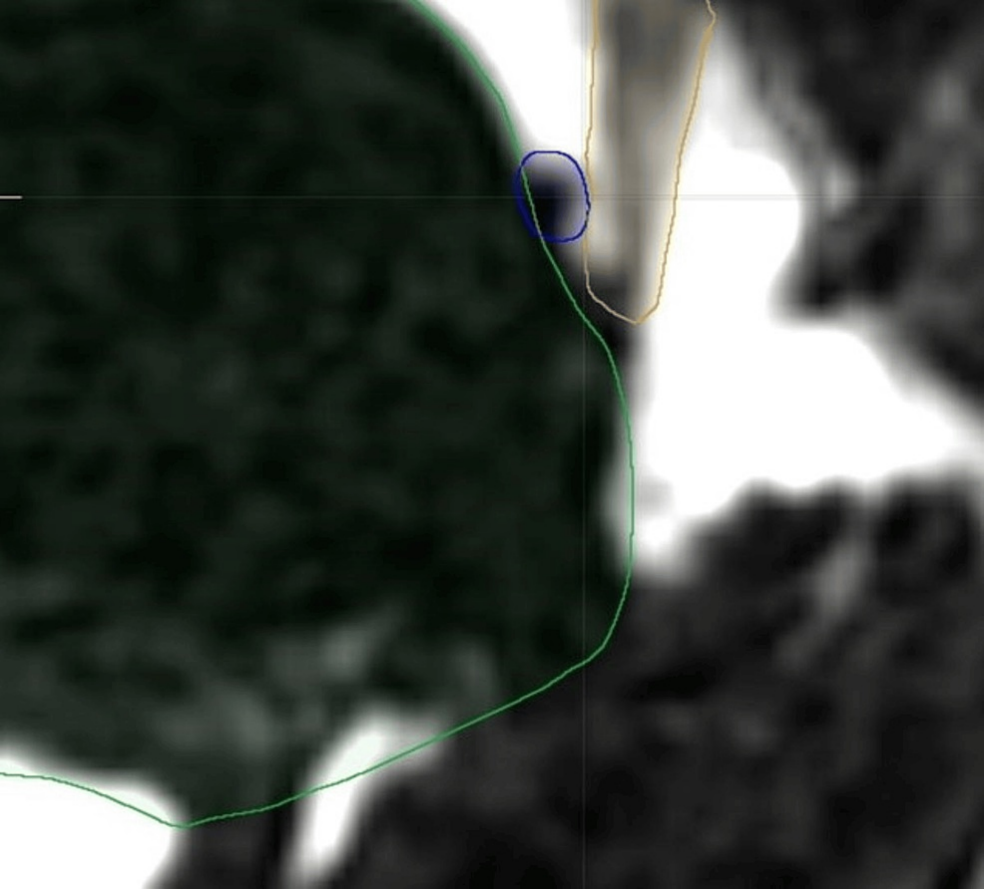
Segmentation of transverse axial MRI-CISS sequence of a 63-year-old female patient with left-sided trigeminal neuralgia. In this image, the brainstem is marked in green color, the left trigeminal nerve in brown color, and the causative venous vessel in blue color. MRI: magnetic resonance imaging; CISS: constructive interference in steady state

In this study, a 63-year-old female patient with intractable left-sided trigeminal neuralgia in the maxillary and mandibular divisions underwent MVD. The preoperative high-resolution MRI data were segmented semiautomatically (manual segmentation of cranial nerves and vessels, automatic segmentation of the cerebrospinal fluid space) to highlight the cranial nerves, brainstem, and corresponding vascular structures for the application of neuronavigation and AR (Figure 1). After image fusion of the essential data sets, the interesting neurovascular regions (vessels, nerves, brainstem) were outlined manually (Brainlab Cranial Navigation, Munich, Germany) for the potential use of AR to enhance the surgeon's visual field during MVD.

The patient was positioned supine with the head fixed in a head holder clamp and the head turned to the contralateral side [7].

A reference array was attached on the right side for left-sided MVD. Head-up displays, showing the AR data, were integrated into the surgical microscope (Kinevo 900, Zeiss, Oberkochen, Germany). The registration array, which was attached to the surgical microscope, was tracked by optical navigation, which took about seven minutes. All microsurgically relevant structures (cranial nerves, vessel loops, brainstem parenchyma) were delineated using the AR display in the microscope.

The causative vessel in the treated patient was a venous branch of the superior petrosal vein, which was successfully mobilized from the trigeminal root entry zone. Teflon was used as interponation material. The intraoperative neuromonitoring values including brainstem evoked potentials were regular. The patient was pain-free in the postoperative period, the pain medication was immediately tapered after surgery, and the patient was discharged symptom-free on postoperative day 5.

## Discussion

The educative aspect of preoperative surgical planning by virtual reality certainly represents a sensible tool [1-2]. 3D visualization in MVD may alleviate complex microsurgical orientation at the ventral and ventrolateral surface of the brainstem. AR especially can assist in the education and training of young surgeons allowing for practice outside the operating room. 

With the multiple picture-in-picture fade-in, the clear anatomical, microsurgical view can get affected. However, the fading of intraoperative AR into the surgical microscope requires additional time and personal assistance, although the amount and selection of visualized objects can be adjusted [14].

MVD is an anatomically clear microsurgical procedure, where neuronavigation from a personal point of view might support a better estimation of the courses of the transverse and sigmoid sinuses for minimizing the craniotomy site. 

In the classical approach, a thorough planning of the surgical procedure is highly recommended.

The implementation of a prospective study dealing with microscope-based AR in MVD is needed, in which this technique still will remain questionable in this procedure.

## Conclusions

AR assistance in MVD may alleviate complex microsurgical orientation at the ventral and ventrolateral surface of the brainstem in the preoperative surgical planning. The intraoperative integration into the microscope is a time-consuming factor and should be well-considered in non-tumorous and non-aneurysmatic vascular operations such as MVD.
